# Examining the Client Experience of Digital Tools in Blended Care Therapy: Qualitative Interview Study

**DOI:** 10.2196/68249

**Published:** 2025-04-02

**Authors:** Emily G Lattie, Miranda Beltzer, Alethea Varra, Connie E Chen, Anita Lungu

**Affiliations:** 1 Lyra Health Burlingame, CA United States

**Keywords:** anxiety, depression, blended care therapy, mental health care, digital mental health, digital tools, qualitative interviews, Lyra Care Therapy, video lesson, symptom assessment, written exercise, thematic analysis, LCT model, therapeutic value, client experience

## Abstract

**Background:**

Lyra Health’s short-term blended care therapy model, Lyra Care Therapy (LCT), has demonstrated effectiveness at scale. In LCT, clients participate in synchronous telehealth sessions and asynchronous guided practice sessions, in which they are provided with digital tools to reinforce key concepts and skills. These digital tools include animated video lessons that use storytelling to show characters learning and implementing new skills from therapy, written psychoeducational materials, interactive exercises that prompt reflection and skills practice, symptom assessments, and messaging with therapists. Past research on LCT found that time spent in therapy sessions and viewing digital video lessons predicts improvements in depression and anxiety symptoms.

**Objective:**

This study aims to explore the client experience of LCT digital tools and to understand clients’ perceived benefits and challenges of using digital tools while in LCT.

**Methods:**

In total, 12 ethnically and racially diverse adults (5 male, 6 female, and 1 pangender) who had graduated from LCT in the previous 4 months participated in semistructured interviews. Interviews focused on experiences with the digital components of LCT (ie, video lessons, symptom assessments, and written exercises). Transcripts were analyzed using thematic analytic methods to determine the benefits and challenges associated with components of the LCT model.

**Results:**

In total, 3 primary themes were generated through thematic analysis. These themes centered around supporting knowledge and skill development, the benefits and challenges associated with the range of digital tools, and the combination of flexibility and accountability promoting positive change. First, we summarize the ways in which guided practice sessions allow clients to develop additional knowledge and learn skills related to their mental health and well-being. Then, we describe how the range of digital tools included in LCT presents different benefits and challenges for clients. Finally, we discuss how flexibility inherent in having both synchronous and asynchronous sessions, along with the accountability from a provider, encourages clients to continue to practice skills related to their mental health and well-being.

**Conclusions:**

Results provide insights into the unique contributions of different components of the LCT model on therapeutic gains. While perceived time constraints and content preferences can impact engagement with digital tools, overall the digital tools were perceived as carrying significant value for participants in the LCT program.

## Introduction

Blended care therapy programs, which combine synchronous face-to-face therapy sessions with asynchronous therapeutic digital content, can offer the benefits of both traditional face-to-face and more novel internet-based mental health services [1-3]. Blended care therapy programs can differ in the degree to which they focus on the synchronous and asynchronous components. Some programs have focused primarily on adding face-to-face contact to digital interventions [4-6], while other programs have more deeply integrated the synchronous and asynchronous components of therapy to create new models of care delivery [7,8]. These integrated blended care therapy models typically address many of the barriers to engaging with traditionally delivered in-person care, including transportation, geography, mobility, and time constraints [9,10]. These blended care therapy models also address many of the barriers to engaging with entirely asynchronous internet-based mental health interventions, such as low perceived relevance of the intervention tools to one’s own life, a lack of motivation to consistently use digital tools, and a poor user experience with digital tools [11,12].

Lyra Care Therapy (LCT) is Lyra Health’s short-term blended care therapy model that is offered as an employee mental health benefit. LCT is an integrated blended care therapy model that has demonstrated effectiveness at scale for treating both depression and anxiety across diverse racial and ethnic groups [13,14]. This is a flexible model of blended care therapy and treats a wide variety of clinical presentations. In a sample of over 33,000 clients who received LCT in the United States, 86.6% of clients met criteria for clinical improvement by the end of care. Further, this recent research demonstrates that completing digital lessons and exercises and receiving provider feedback messages was associated with greater improvements in client depression and anxiety symptoms, beyond the effect of therapy sessions [14].

In LCT, clients participate in synchronous telehealth therapy sessions and in asynchronous guided practice sessions. The average length of an LCT episode is 6 synchronous therapy sessions over 8 weeks [14]. Consistent with best practices in mental health care, Lyra Care therapists are trained in and receive ongoing supervision and consultation on evidence-based mental health care [15,16]. Synchronous telehealth therapy sessions, in which therapists meet with clients on a secure video platform that is compliant with the Health Insurance Portability and Accountability Act, last for 50 minutes and typically occur weekly or biweekly. During these synchronous therapy sessions, therapists and clients establish goals for treatment, work together on developing and practicing strategies and skills, and monitor progress toward those treatment goals. Therapists also use time in these sessions to introduce the content that they are recommending the client to use during the guided practice sessions. As a flexible provider-led model of blended care, the recommended digital tools are selected in a collaborative way based on the content of the session, the client's goals, case conceptualization, and anticipated challenges during the following week; then the provider gets commitment from the client to do the practice.

Guided practice sessions take place on the digital therapy platform, which clients can access on a web browser or via the Lyra Health app. The “guided” aspect of guided practice sessions refers to therapists guiding clients to asynchronous tools relevant to their care, sending messages in between sessions, and providing asynchronous feedback on the client’s progress as the client completes between-session activities. The “practice sessions” aspect refers to the asynchronous skills building and practice that happen when clients engage with these tools between sessions. This content is accessible asynchronously at any time of day or night. These digital tools were developed by Lyra Health based on transdiagnostic treatment approaches, including the Unified Protocol [17], acceptance and commitment therapy [18], and dialectical behavior therapy [19]. After each synchronous therapy session, therapists assign digital tools to their clients to reinforce key concepts and skills related to what is being worked on in treatment. These digital tools include animated video lessons that use storytelling to show characters learning and implementing new skills from therapy, written psychoeducational materials, interactive exercises that prompt reflection and skills practice, symptom assessments, and messaging with therapists. The digital tool library has grown over time, with new video lessons, exercises, and guides added on an ongoing basis. As the client engages with the digital tools, the therapist receives alerts and is able to provide asynchronous support through in-platform commenting and messaging. In a recent analysis of over 33,000 clients who completed LCT, participants viewed a median of 6.0 (IQR 3.0-9.0) digital lessons, completed 6.0 (IQR 2.0-12.0) exercises, and exchanged 16.0 (IQR 9.0-28.0) direct messages with their providers during guided practice sessions [14]. This model supports the practice of skills outside of synchronous telehealth sessions and helps support therapist fidelity to evidence-based practices.

Past research on LCT found that time spent in therapy sessions, time spent viewing digital video lessons, time spent completing exercises, and time spent messaging with the therapist all predict improvements in depression and anxiety symptoms [14,20]. These findings indicate unique statistically significant and clinically significant benefits for clients engaging in both the synchronous telehealth sessions and in the asynchronous guided practice sessions.

However, little is known about the client experience of participating in LCT. For clients to fully engage in mental health services, the services should be optimized to have a strong user experience. Qualitative research on a slightly different model of blended care therapy, which included a greater average number of telehealth sessions (an average of 21.96 sessions relative to our average of 6 sessions) and a different model of between-session practice assignments (consisting of text-based lessons, exercises, and testimonials, with small interactive elements such as audio and video clips), found that clients were generally satisfied with all components of the blended care therapy model but sometimes felt overwhelmed by the digital tools [7]. Identifying the client-perceived value of and challenges associated with specific components of blended care services is essential to understanding and designing more usable, enjoyable, and effective methods of delivering mental health care. This study explores the client experience of digital tools while in LCT and aims to understand the perceived benefits and challenges of using digital tools while participating in LCT.

## Methods

### Overview

We conducted a semistructured interview study to explore the client experience of using digital tools while in LCT and to understand our client’s perceived benefits and challenges for engaging with these tools.

### Participants

To ensure that potential participants had familiarity with LCT digital tools and would be able to reflect on their experiences in LCT, we sent a recruitment email to Lyra clients who had graduated from LCT in the prior 4 months, were at least 18 years of age, and had completed at least 50% of digital assignments (ie, video lessons, exercises, and guides) during their course of therapy. LCT is available to individuals as an employer-sponsored mental health benefit, Lyra Health, offered by Lyra Clinical Associates. Potential participants were invited to complete a short screening survey to confirm their interest in participating and to provide demographic information. Invitations to schedule an interview were sent to participants, along with a copy of the institutional review board–approved consent document.

### Ethical Considerations

All study procedures were approved by the Western-Copernicus Group Institutional Review Board (approval number: WCG IRB Protocol #20232618) prior to enrolling participants. Participants were sent a copy of the institutional review board–approved consent document prior to their scheduled interview. At the beginning of the interview appointment time, the interviewer reviewed the consent form, and participants provided verbal consent. After the interviews, participants were compensated for their time with a US $75 gift card. Interviews were recorded for transcription purposes, and the recordings were deleted after transcription was completed. All quotations presented in this manuscript have been deidentified.

### Study Procedures

We interviewed a total of 12 participants. Interviews lasted approximately 60 minutes and were conducted over Zoom by the lead author. After verbal consent was obtained, the interview began and was recorded for transcription purposes. During these semistructured interviews, participants were prompted to reflect on their experiences in LCT, including the impact that the digital tools had on their lives and how different types of digital tools may have helped or hindered their progress in psychotherapy. Example questions from the interview guide include “How would you describe the experience of having digital content to work on in between sessions? What was helpful? What was unhelpful?” and “What components of the platform were most helpful to you in making progress in therapy?”

### Positionality Statement

The authors all have training as clinical psychologists or physicians and have experience in the design, development, and implementation of technology-enabled mental health services. The majority of the authors have experience in delivering evidence-based psychotherapy to individuals across the lifespan and drew on these professional experiences while designing the study and interpreting the data. Throughout the analysis, the authors took their own positionality and identities into account while analyzing study data.

### Analysis

Transcripts were analyzed in Dedoose [21] using a codebook thematic analytic approach [22]. The 6 steps of analysis, outlined by Braun and Clarke [23], were followed, in which the lead author familiarized themself with the data prior to generating a preliminary codebook used to organize the initial analysis of the data. Reflexive practices were used to iteratively develop themes, and the themes were reviewed, defined, and presented in this paper. Analytic decisions were documented throughout the research process. Because interrater reliability rests on the assumption that meaning is fixed within the data, this analytic approach does not recommend using formal interrater reliability metrics [22].

## Results

### Overview

A total of 12 individuals participated in these interviews. As seen in Table 1, participants held diverse social and cultural identities.

In total, 3 primary themes were generated through thematic analysis. These themes centered around supporting knowledge and skill development, the benefits and challenges associated with the range of digital tools, and the combination of flexibility and accountability promoting positive change. First, we summarize the ways in which guided practice sessions allowed clients to develop knowledge and learn skills related to their mental health and well-being. Then, we describe how the range of digital tools included in LCT presents different benefits and challenges for clients. Finally, we discuss how flexibility inherent in having both synchronous and asynchronous sessions, along with the accountability from a provider, encourages clients to continue to practice skills related to their mental health and well-being.

**Table 1:** Participant demographics.

Gender			Participants, n
Male			5
Female			6
Pangender			1
**Age (years)**			
	18-24	1	
	25-34	4	
	35-44	2	
	45-54	2	
	≥55	2	
**Race or ethnicity**			
	Black	3	
	South Asian	2	
	East Asian	1	
	Hispanic or Latino	1	
	White	5	
**Education**			
	Vocational school	2	
	Some college	2	
	Bachelor’s degree	6	
	Master’s degree	2	
**Household annual income (in US $)**			
	Less than $20,000	1	
	$20,000-$34,000	1	
	$35,000-$49,000	2	
	$50,000-$74,000	2	
	$75,000-$99,000	2	
	More than $100,000	3	

### The Addition of Asynchronous Guided Practice Sessions Allows for the Development of Both Knowledge and Skills

Gaining knowledge and developing skills are central to making gains in psychotherapy. The combination of synchronous therapy sessions with a mental health provider and asynchronous guided practice sessions was viewed by many participants as essential to their progress in LCT. While many of the participants were familiar with synchronous therapy sessions, either from their own past treatment experiences or general knowledge of what “therapy” typically entails, the concept of having asynchronous guided practice sessions was new to most participants and appeared key in supporting the development of knowledge and skills. Overall, these asynchronous sessions supported clients in using the synchronous sessions more efficiently and often allowed clients and therapists to communicate more effectively about issues related to the care episode.

Participants shared ways in which the asynchronous guided practice sessions were integrated into their lives and how they helped to identify areas for growth and to provide support for both knowledge acquisition and skills practice. Pt 6 reflected:

I got a video to learn about the approach with cognitive behavioral therapy with my particular therapist and I was sold on that completely. The tools you're learning you then apply in your daily life. I liked the hybrid learning or the flip learning where you do work outside the classroom so when you come to therapy, you can talk about it. And for me, it's important to go back to them outside of that 45 min because if not, we would waste time during my session.

This participant had a background in education and drew these favorable parallels to the increasingly popular flipped classroom pedagogy in which active class time is used for working through exercises and discussing concepts that were learned outside of the classroom. By completing exercises asynchronously, the participant felt that subsequent sessions were more efficient because they could use the time to talk through their progress and any challenges that arose between sessions.

Many participants in the sample had past experiences with receiving mental health care in which knowledge and skill development felt less supported. Pt 5 reflected:

Previously, I had another therapist on a different platform. I had an opportunity to meet with her very regularly and while it was helpful, I did not get the same benefits that I think I did from using Lyra because my therapist with Lyra would issue me little assignments, like videos, that often spoke to situations that I was in personally.

Among participants who reflected on their past experiences in therapy, there were particularly favorable attitudes towards the model of blended care therapy in which asynchronous guided practice sessions supported the clients in making specific treatment gains, often in a more consolidated amount of time. As Pt 12 commented:

Whenever I’ve done therapy without tools like this, it’s taken me a couple of iterations to get to the point. With the tools, I get to think faster.

This notion of “thinking faster,” supported by guided practice session digital tools, appears to translate into more active and engaged practice of therapeutic skills and a quicker time to symptom relief.

Participants commented both on how the use of digital tools in guided practice sessions helped them reflect more deeply and on how therapist review of their digital tools assignments allowed for the therapist and client to communicate more effectively about presenting problems and concerns. As Pt 1 reflected:

So I ended up being assigned the journal… I started going in and just writing a little more and I didn't realize I hadn't truly opened up to the therapist assigned to me. When I started to journal, she learned more. She was like, “we never talked about this. You've never talked about this in sessions”

Here, we see that the guided practice sessions allow for clients to practice skills being taught in therapy and also allow for clients to communicate with their therapists in an enhanced way. Through this enhanced communication, therapists can comment and reinforce or explore new topic areas with their clients. This leads to a more deeply shared understanding and to more personalized approaches to structuring subsequent synchronous therapy sessions.

Pt 5 went on to comment:

It was probably the most effective thing that I've ever had. I appreciated it a lot like, yes, my therapist was amazing. She did a really great job with helping me a lot, but having those videos along with her support and her guidance, I feel really helped me to make the progress that I have today.

Multiple participants echoed this belief that the provider-led selection of the digital tools was critical to their treatment success and that the personalization offered the advantage of digital tools being perceived as highly relevant to the participant and their presenting problems and goals for therapy. It appears that digital tools alone are unlikely to support the reported level of knowledge and skills development. Rather, the combination of synchronous telehealth sessions and the digital tools embedded within asynchronous guided practice sessions was found to be of benefit here.

### The Range of Available Digital Tools Presents Different Benefits and Challenges for Clients

Overall, digital tools appeared to support the acquisition of knowledge and support the practice of skills, while also helping to build a common language between clients and their therapist. In line with what is known about how different individuals will respond to different treatment components in different ways [24], we observed that the various digital tools embedded in LCT each presented their own benefits and challenges for clients. These benefits and challenges were discussed in the context of having the therapist select digital tools based on intended relevancy to the client. Within this theme, we identify factors that contribute to both the strengths and weaknesses of different types of digital tools within blended care therapy.

### Video Lessons

Video lessons (ie, animated videos featuring stories of characters as they face mental health challenges and work with a therapist to learn skills to address those challenges) were the most popular type of digital tool with participants in this study. The majority of participants described favorable ways in which the lesson content was relatable. In addition to videos teaching therapeutic concepts and skills, they served a valuable function in normalizing the experience of a range of mental health concerns. Multiple participants reflected on how video lessons helped deepen their understanding of skills and strategies that were discussed in therapy sessions. For example, Pt 6 noted:

The videos allowed you to see glimpses of what was happening in [the character’s] life, but then they would use strategies to work through in the moment just like what I was doing with [provider name]. It was not just teaching, but it was also practicing the strategies which was helpful because it creates the framework right of how to actually implement it, think about it and process it, which creates a roadmap.

Storytelling, as evident in these video lessons, appears to be a vital component for supporting the learning acquisition process.

Similarly, Pt 10 highlighted:

I think the videos helped a lot. It puts things on a more personal and a less straight, clinical level… I remember actually watching some of the first videos that were an introduction to depression and anxiety and it walks you through the different effects that it can have on someone and the different ways it can manifest. That part really resonated with me and I really understood it. It made me feel less crazy and instead of having something wrong, I understood this disconnect between my thoughts and my emotional reactions and how it is something that I can work through. It's absolutely something that other people also deal with and that definitely helped a lot.

The ability of lessons to normalize common mental health issues appeared to help this participant overcome self-stigma related to his symptoms and become more motivated to make changes.

Providing video lessons without support or guidance may be less useful than when integrated into a blended care therapy model. Pt 5 commented:

I appreciated that in tandem with me venting everything under the sun to her, she was also able to assign me things where if I brought up a very particular problem, she's like, “Oh, this sounds a lot like what you're going through. I want you to take a look at this and to make sure that you're actually paying attention.”

This highlighted how the guidance therapists provide around video lessons supports therapy clients in learning new practices that are in line with what is being discussed in therapy sessions. The support of asynchronous learning and skill practice allowed for the client to work toward their therapeutic goals within the context of a supportive relationship with an expert clinician.

However, video lessons presented some challenges. Some participants reported that it was challenging to find time to view the videos, which are typically 8-10 minutes long. Although many participants found that the video lessons fit well into their lives, a small portion of study participants reported that it was challenging to keep up with the video lessons (and other digital tools) due to the time commitment required outside of synchronous therapy sessions. For example, Pt 9 described having some physical health issues emerge and needing to spend significant time and energy navigating the health care system. They reflected that:

medical stuff was just tacked on top of it, so remembering to do the check-ins and watch all the videos was derailed and I didn’t always keep up with it.

For some people, dedicating 50 minutes every week or two to synchronous therapy sessions feels like the maximum amount of time that they can dedicate to mental health care.

Participants varied in how much they related to characters presented in the video lessons. For example, a college-aged man reflected that a specific lesson featuring a middle-aged woman character, who was overwhelmed with messes that her husband and child were leaving around the home, was particularly relatable because:

this kind of thing is absolutely something that happens in most people's lives whether it's a mom dealing with a mess or whether it's me dealing with roommates and a mess. I definitely think it can create some trouble in thinking clearly and can create some very angry and tense emotions. Having that reminder that I can walk through it using the Lyra platform is very helpful.

However, a middle-aged woman participant reflected on the same digital lesson as less engaging, noting:

I didn’t relate to it because I don't have kids. There were other videos featuring kids, a husband, and work stress and my situation is kind of different because I have a fiance, we don’t have kids, and my work doesn’t necessarily stress me out.

Having relatable characters featured in the video lessons appears to increase clients’ ability to empathize with and learn from the lessons, and further examination of factors that increase and decrease relatability will be fruitful for the continued development of characters and storylines for video lessons.

### Digital Exercises

Some participants found it challenging to write about their thoughts, feelings, and experiences in digital exercises, though with encouragement from their providers, they found these exercises helped them feel better. While a few of the participants reflected sentiments such as “writing isn’t my thing,” participants also described ways in which their therapists were able to tailor the written assignments to meet their needs. For example, Pt 6 reflected:

I get caught in needing to write it perfectly… [provider name] made it really clear that it doesn't matter and to make bullet points in a section and we can work with it. She used it as an opportunity to say, “what do you think it's about writing or putting your things down?” So it was good to be able to name it and process it. But it is also attached to some of the challenges.

This discomfort with writing down thoughts or experiences was echoed by other clients who did not initially find written exercises to be helpful. Pt 4 continued to reflect on this point by noting:

When I did write the little bits that I did, I did feel better. It wasn't too pushy. It was just like you said, just a little bit of encouragement.

For participants like these ones, the guidance and support of the therapist appeared essential for them to be able to engage with, and subsequently benefit from, the written exercises.

### Symptom Assessments

While in LCT, clients are prompted to complete symptom assessments prior to each session. This practice is in line with measurement-based care principles and allows both the therapist and the client to track changes in symptoms and adjust treatment as necessary. Some participants found the weekly symptom assessments to be helpful and motivating from the beginning of therapy. For example, participants who had past experiences in therapy without routine symptom assessments, such as Pt 9, stated:

The most useful to me was the check-ins to see where I'm at and see where the progress was over time. I liked checking in to see where I was at and to see progress.

However, some participants did not initially value these assessments but grew to appreciate them over time. As Pt 6 highlighted:

I was answering the same questions over and over again. But then, all of the sudden my responses were starting to change. It felt affirming because at first, I was just producing the same answers and then they changed! It showed progress for myself.

While symptom assessments felt repetitive and tedious for some, the vast majority of participants appreciated the feedback that symptom assessment provided in terms of progress tracking.

### Flexibility and Accountability Encourage Continued Skills Practice

Evidence-based short-term therapy models, such as the Lyra Care model, typically focus on developing and then practicing new skills or strategies to reduce suffering and improve well-being. Clients are prompted to set goals for treatment at the onset of a course of therapy, and the client and therapist work together to support progress toward those goals. As clients make progress on their goals, they work toward graduating or taking a break from therapy. This is marked by a client being ready to stop having regularly scheduled therapy sessions and to manage any remaining symptoms independently or with the support of their social network. In these short-term therapy models, therapy is conceptualized as a time to learn and apply skills and to begin generalizing those skills. When someone graduates from therapy, they are encouraged to continue doing the things that work well for them and to have a plan for how they will address new challenges moving forward. The combination of flexibility and accountability woven into this model of blended care therapy, and supported by the available digital tools, appears to promote continued practice of therapeutic skills.

Through these interviews, we observed that the digital content available through the guided practice sessions provided durable opportunities for reflection and discovery. Digital tools could prompt clients to reflect on their current experiences, identify their current emotions and thoughts, and discover both factors contributing to their current emotional state and strategies that they could use to feel better. Convenience matters when examining how a digital tool can be used while in therapy and how it might be used after graduation. Several participants reported that the written exercises were important for practicing skills and keeping the momentum going in between sessions. As Pt 10 commented:

I got a lot more comfortable with taking out my phone when I started having an issue or even just making a mental note of what I was going to put in a thought log or something of that nature. It's extremely helpful as an in the moment tool while also being something that is discrete enough that I could take out my phone any time of the day, anywhere I was, and just seem like I was texting where in reality I was, I was working through whatever stress or anxious thoughts I was having, so that definitely became a very helpful tool.

Having supportive tools almost always available to use can be key for integrating and applying the skills taught in LCT to diverse situations in one’s life.

While a plethora of digital mental health tools are available, many individuals report difficulty in finding high-quality digital mental health tools and are quick to disengage from using such tools [25,26]. Here, we observed that the guidance from and accountability provided by the blended care therapist helped participants learn how specific tools could be useful to and usable for them. Tools were typically introduced during synchronous telehealth sessions, and then follow-up support and recommendations were given during guided practice sessions. Pt 8 noted:

my therapist did a good job of marketing the tools to me of this being something you can do if you’re feeling like this, and giving me examples of when even would be good times to even use it. So, when I would be experiencing a certain feeling that we discussed in therapy, I would be reminded of my therapist telling me that I should do this when I experience this, and that would lead me to go onto the platform and try an activity.

Participants also spoke of how automated prompts from the system prompted greater accountability to engage. Pt 5 reflected:

During my time with my therapist, I would get, like, little email reminders or text message reminders that were prompting me to make sure that I get my assignments or my weekly health assessments done prior to my next session. I found that really helpful.

This support received from both the therapist and the automated system prompted clients to experiment with tools during an episode of care and learn how the tools can benefit them, which can then facilitate ongoing habitual practices.

While video lessons were particularly popular for clients while they were in care with their therapist and supported the learning of new skills, written practices were often carried forward after graduating from a course of blended care therapy as a means of continued skill practice. For example, Pt 8 noted:

Now, I have a journal and even though it's not necessarily the Lyra exercise itself, it had components that I remember from it. When I was really anxious the other day about some random thing, I just opened up a note on my phone and started challenging my thoughts.

This participant described one of the primary goals of short-term CBT—that they were able to better recognize their emotional experiences and quickly apply an evidence-based strategy (cognitive restructuring) to proactively manage their experience.

## Discussion

### Principal Findings

Results of this study support the previously observed therapeutic value of digital video lessons in LCT and provide insights into the unique contributions of different components of the LCT model on therapeutic gains. While perceived time constraints and content preferences can impact engagement with digital tools, overall the digital tools were perceived as carrying significant value while participants were clients in LCT. The combination of synchronous telehealth sessions and asynchronous guided practice sessions both helped clients learn new skills that were personalized to their current needs and helped clients apply these skills in their lives through between-session practice and feedback from therapists.

Asynchronous and synchronous components of this blended care model (ie, LCT) worked together to produce client-reported benefits. The clinically important elements of this treatment model seem to be (1) the therapist selecting relevant skills, (2) the therapist introducing skills in session, (3) the client completing between-session practice, (4) the therapist reviewing and commenting on between-sessions practices, and (5) reflection in the subsequent synchronous session. Guided practice sessions appear to enhance synchronous therapy sessions by helping facilitate ongoing communication between therapist and client and supporting skills acquisition and practice both in and out of sessions. Together, this model appears to allow clients to make clinical progress more quickly than they have seen in traditional models of psychotherapy.

Past quantitative analyses have demonstrated the value of video lesson viewing on clinical outcomes. These qualitative analyses further support the value of video lessons. Storytelling, embedded in these lessons, appears to be important in normalizing a range of experiences. Storytelling in digital mental health interventions is increasingly being recognized as clinically valuable. Narrative-based mental lessons support clients in reflecting on their own patterns and help them to feel a sense of connection to similar others as they apply the concepts to their own lives [27]. Past research has found that satisfaction with client stories embedded in internet-delivered cognitive behavioral therapy to be related to both treatment satisfaction and decreases in anxiety symptom severity [28]. Many clients in this study found the video lessons normalized experiences and that the characters and storylines were relatable, sometimes in surprising ways. However, some clients had more difficulty relating to the characters. Given that relatability appears to support engagement in the guided practice session process, this opens future directions for improving the relatability of digital lesson content. Additional research should be conducted to better understand which characteristics are most important for relatability and how relatability of characters relates to effectiveness of between-session practice. Additional training for therapists on how to frame the between-session practice may also be warranted to better support clients in developing their ability to relate to and engage with the important elements of the lesson.

At its core, LCT is an evidence-based short-term therapy model. While short-term models of therapy have strong research evidence for reducing symptoms of mental ill-health [29,30], not all individuals are initially comfortable with or confident in the approach of short-term therapy. Between-session practice helps clients build structure and self-efficacy in using skills that prepares them for a confident graduation. A large body of literature demonstrates that completing therapy homework relates to more positive treatment outcomes [31,32]. Here, we observed that the structure of skill practice outside of synchronous telehealth sessions allowed clients to figure out what skills work for them and in what situations those skills can be used. Participants in this study reflected on ways in which they figured out how to practice skills in real time, such as on their smartphones or by writing in a notebook. These skill-learning experiences are supported both with guided practice sessions and troubleshooting during synchronous sessions with the therapist. As digital tools were identified as being a helpful component of the LCT process with specific benefits of supporting work toward therapy graduation, additional work should be done to determine how to best support the transition to self-care with digital tools after graduation. Barriers to engaging in between-session practice included both time constraints and content preferences (eg, video length, preferences for reading or listening). Future work should continue to explore how clients would respond to different formats of between-session digital content.

### Limitations and Additional Future Directions

The qualitative nature of this study is, in many ways, a strength. Qualitative research allows for deeper understanding of our client experience by honing in on how research participants experienced different components of LCT. However, the methods employed in this study do not support the quantification of experiences [33], and qualitative research typically does not aim to be generalizable across populations. Thus, the experiences detailed in this manuscript are unlikely to be representative of all experiences that one may have in LCT. Historically, research on technology-enabled mental health interventions has been conducted on largely homogenous samples as White individuals, and particularly White young to middle-age adult women, are typically over-represented in research trials [34,35]. While our study sample was diverse in regards to race, ethnicity, gender, age, and income, the participants may not be representative of all clients who engage with blended care services. We intentionally selected a sample of clients who had been engaged with digital content while in therapy (by having completed at least 50% of assignments)—this decision was made to maximize the likelihood that participants would be able to meaningfully reflect on the utility of the different treatment components and the full model of LCT. We cannot report on the exact digital tools each participant in the study used during their course of therapy as their engagement in therapy was separate from their engagement with this qualitative research study. Clients in LCT are not “required” to complete digital content to progress in therapy, and this sample does not represent individuals who did not deeply engage with the digital tools. Additionally, therapy is a dyadic process involving both a client and a therapist, and both sides of this process, as well as the interactions between the client and therapist, should be examined [36]. This study is reflective only of the client experience of digital tools while in LCT and the client-perceived benefits and challenges of using digital tools while participating in LCT. Future work will examine the therapist's experience of delivering this type of blended care.

### Conclusions

The between-session digital content embedded in short-term blended care therapy programs, such as LCT, can keep therapy clients engaged in skill development outside of sessions and can drive therapeutic gains. This model of care has demonstrated clinical effectiveness at scale in past quantitative studies and can support clients in developing mental health and coping skills in a relatively limited treatment period (average of 6 sessions over 8 weeks [1,14]). These qualitative results support the previously observed therapeutic value of digital video lessons in LCT and provide insights into the unique contributions of different components of the LCT model on therapeutic gains. As we continue to work toward increasing access to evidence-based mental health care, blended care therapy programs can be considered as a viable method for meeting client desires to work synchronously with a mental health provider while also offering the benefits of asynchronous care focused on skill development.

## Abbreviations

LCT: Lyra Care Therapy

## Data Availability

Given the small dataset, interview data are not publicly available to ensure participants’ privacy. For questions about the dataset, contact the corresponding author.
